# Lactylation at the crossroads: Bridging metabolic rewiring and ferroptotic vulnerability in cancer

**DOI:** 10.1016/j.redox.2026.104341

**Published:** 2026-08-11

**Authors:** Yali Liu, Qiang Yang, Xiaoqiang Wang, Hai Zhao

**Affiliations:** a Department of Neurosurgery, Lanzhou University Second Hospital, No. 82 Cuiyingmen, Chengguan District, Lanzhou City, Gansu, 730030, China; b Department of Neurosurgery, The Affiliated Hospital of Qingdao University, No. 16 Jiangsu Road, Qingdao, Shandong, 266005, China

**Keywords:** Histone lactylation, Ferroptosis, *Warburg* effect, Epigenetic remodeling, Tumor microenvironment, Metabolic vulnerability

## Abstract

Metabolic reprogramming and the evasion of regulated cell death are two canonical hallmarks of cancer, yet the mechanistic threads connecting these distinct biological phenomena remain incompletely understood. The “Warburg effect,” characterized by robust aerobic glycolysis, results in the massive accumulation of lactate. Once dismissed as a metabolic waste product, lactate has recently emerged as a pivotal signaling molecule and an epigenetic precursor for lysine lactylation (Kla), a novel post-translational modification that fundamentally reshapes the chromatin landscape. Concurrently, ferroptosis—an iron-dependent form of non-apoptotic cell death driven by unrestrained lipid peroxidation—has garnered intense interest as a therapeutic vulnerability in therapy-resistant tumors. However, a paradoxical observation persists highly glycolytic tumors, despite generating abundant ROS (reactive oxygen species), often display intrinsic resistance to ferroptosis. In this review, we propose that histone and non-histone lactylation serves as the critical “epigenetic bridge” coupling metabolic flux to ferroptosis evasion. We dissect the molecular machinery by which the “Lactate–Kla axis” transcriptionally activates antioxidant defenses (e.g., GPX4, SLC7A11) and remodels the tumor microenvironment to suppress ferroptotic triggers. Furthermore, we delineate how targeting the writers and erasers of lactylation can dismantle this metabolic shield, offering a rational strategy to re-sensitize refractory tumors to ferroptosis-inducing therapies. This synthesis highlights a new frontier in cancer biology where metabolism, epigenetics, and cell fate decisions converge.

## 1 Introduction

The relentless proliferation of malignant cells necessitates a profound rewiring of cellular metabolism. Since *Otto Warburg's* seminal observation nearly a century ago, the shift toward aerobic glycolysis—even in the presence of ample oxygen—has been recognized as a defining feature of the oncogenic state [1]. This metabolic phenotype supports rapid biomass accumulation but comes at the cost of generating profound quantities of lactate. For decades, lactate was viewed merely as a simplified bystander or an acidic waste product of inefficient metabolism [2]. This paradigm was radically overturned by the discovery of Kla, a novel histone post-translational modification derived directly from lactate, which links metabolic status to the epigenetic regulation of gene expression [3]. This finding positioned lactate not as a waste product, but as an active architect of the cancer genome, capable of “locking in” specific transcriptional programs that favor survival and progression [4,5].

Parallel to these metabolic insights, the field of cell death has witnessed a renaissance with the characterization of ferroptosis [[5], [6], [7]]. Distinct from apoptosis, autophagy, and necroptosis, ferroptosis is an iron-dependent form of regulated necrosis driven by the catastrophic accumulation of lipid peroxides within cellular membranes. Because rapidly dividing cancer cells possess an insatiable appetite for iron and generate high levels of metabolic ROS, they should theoretically be exquisitely sensitive to ferroptosis [5]. Yet, clinical reality contradicts this expectation: many aggressive, highly glycolytic tumors exhibit intrinsic resistance to ferroptosis-inducing agents. This paradox suggests the existence of a robust, underlying coupling mechanism where high metabolic flux actively reinforces antioxidant defenses.

In this review, we posit that lactylation is the missing mechanistic link bridging the Warburg effect and ferroptosis resistance. We explore emerging evidence suggesting that the “lactate clock” regulates the ferroptotic threshold by directing the transcriptional activation of key antioxidant systems, such as the System $X_{c}^{-}$/GSH/GPX4 axis (cystine/glutamate antiporter/glutathione/glutathione peroxidase 4), via H3K18la (histone H3 lysine 18 lactylation) and other distinct marks. We further examine how this axis operates beyond the cancer cell, influencing the TME (tumor microenvironment) to create a “cold” niche hostile to ferroptotic surveillance. Finally, we discuss the therapeutic implications of disrupting this “metabolic-epigenetic-death” triad, proposing that decoupling lactylation from antioxidant defense may represent a potent strategy to unleash the full potential of ferroptosis-based cancer therapies.

## 2 The metabolic basis: lactate accumulation and iron handling in the tumor milieu

The cellular decision to undergo ferroptosis or sustain survival is not merely a signaling event but a direct consequence of metabolic flux. To understand how lactylation functions as an epigenetic brake, we must first dissect the colliding metabolic currents of aerobic glycolysis and iron addiction that characterize the malignant state.

### 2.1 The Warburg effect: from ATP generation to lactate saturation

**Fig. 1:**
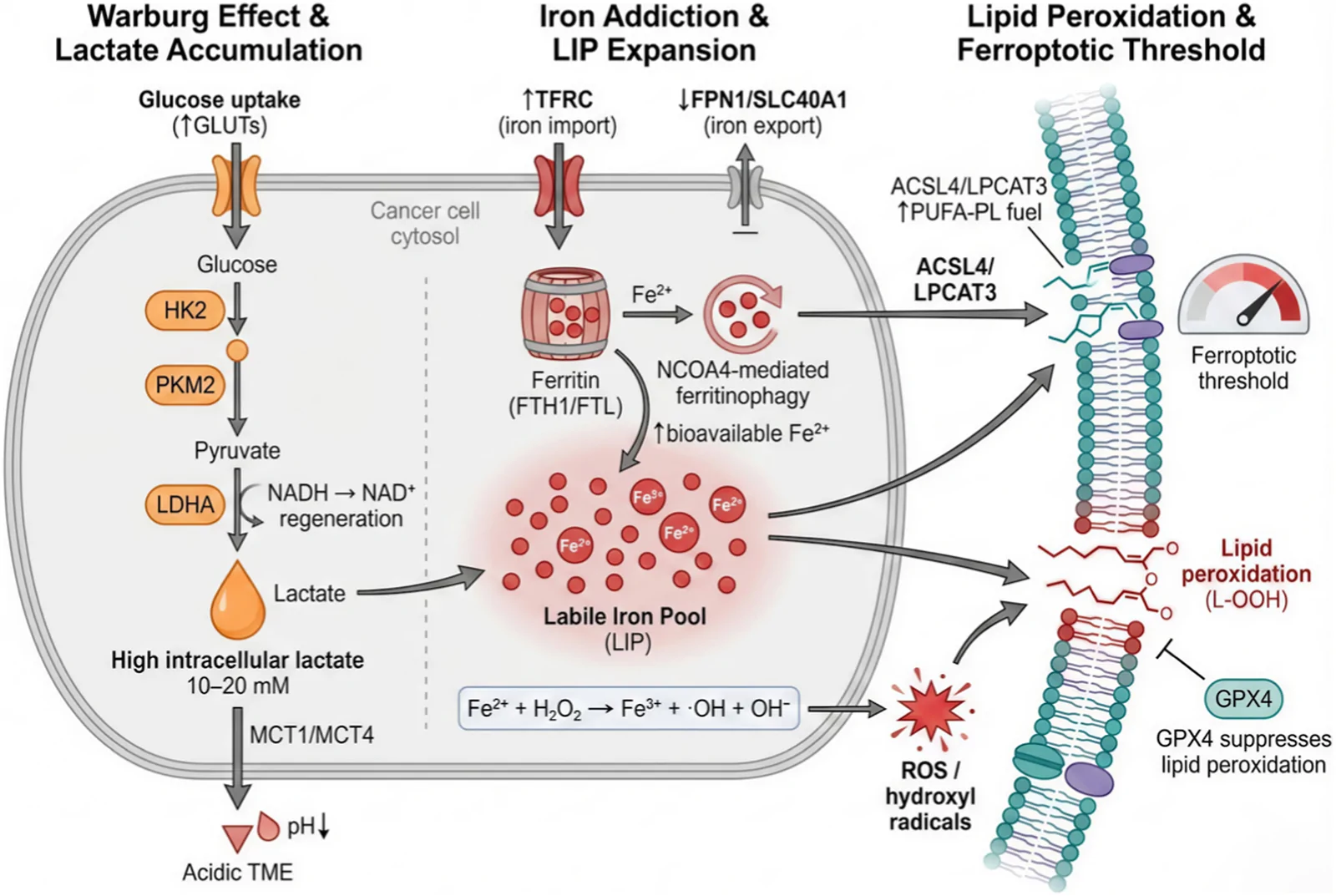
*Warburg effect*-driven lactate accumulation and iron addiction converge to elevate ferroptotic pressure. **(Left panel)** The *Warburg effect* is characterized by increased glucose uptake via GLUT transporters. Glycolytic enzymes HK2 and PKM2 process glucose into pyruvate. LDHA converts pyruvate to lactate, coupled with NADH to NAD + regeneration, resulting in high intracellular lactate accumulation (10–20 mM, orange droplet). Excess lactate is exported via MCT1/MCT4, leading to an acidic TME. **(Center panel)** Iron addiction is driven by upregulated TFRC-mediated iron import and downregulated FPN1/SLC40A1-mediated export. NCOA4-mediated ferritinophagy degrades ferritin (FTH1/FTL storage), releasing bioavailable ferrous iron (Fe^2+^) and expanding the LIP (red circles). Free Fe^2+^ participates in Fenton chemistry with hydrogen peroxide (H_2_O_2_) to generate highly reactive hydroxyl radicals (ROS, red burst icon). **(Right panel)** The convergence of these pathways drives lipid peroxidation at the cell membrane. ACSL4 and LPCAT3 enzymes enrich membrane PUFA-PLs. Both reactive Fe^2+^ from the LIP and ROS attack these PUFAs, causing lipid peroxidation (L-OOH, red lipids) and pushing the cell toward the ferroptotic threshold. GPX4 acts as a negative regulator by suppressing lipid peroxidation. Abbreviations: ACSL4, Acyl-CoA synthetase long-chain family member 4; FPN1/SLC40A1, ferroportin/solute carrier family 40 member 1; FTH1/FTL, ferritin heavy chain/light chain; GPX4, glutathione peroxidase 4; HK2, hexokinase 2; LDHA, lactate dehydrogenase A; LIP, labile iron pool; LPCAT3, lysophosphatidylcholine acyltransferase 3; L-OOH, lipid hydroperoxides; MCT1/4, monocarboxylate transporter 1/4; PKM2, pyruvate kinase M2; PUFA-PLs, polyunsaturated fatty acid-phospholipids; ROS, reactive oxygen species; TFRC, transferrin receptor 1; TME, tumor microenvironment.

The reprogramming of glucose metabolism, known as the *Warburg effect*, involves the preferential utilization of glycolysis over oxidative phosphorylation even under normoxic conditions [8]. While energetically less efficient in terms of ATP yield per glucose molecule, this shift provides rapid generation of biomass precursors and maintains the cellular redox balance required for unrestrained proliferation [8]. Central to this process is the upregulation of glucose transporters and key glycolytic enzymes, including hexokinase 2 (HK2) and pyruvate kinase M2 (PKM2), which funnel carbon flux toward pyruvate [9]. Crucially, instead of entering the mitochondrial tricarboxylic acid (TCA) cycle, most of the pyruvate is converted into lactate-by-lactate dehydrogenase A (LDHA), a reaction coupled with the regeneration of NAD+ from NADH [10]. Consequently, aggressive tumors, including glioblastoma and hepatocellular carcinoma, maintain millimolar intracellular concentrations of lactate, often exceeding 10–20 mM, compared to <2 mM in normal tissues [11] (Fig. 1, Left panel). This lactate is not strictly compartmentalized; it is actively trafficked across the plasma membrane via monocarboxylate transporters (MCT1/4), creating an acidic TME that suppresses immune surveillance [12]. However, recent evidence underscores that a significant pool of intracellular lactate is retained or re-imported to fuel the mitochondrial TCA cycle in neighboring oxidative tumor cells—the “metabolic symbiosis” model—or, more pertinently for this review, shunted toward the nucleus to serve as a substrate for histone modifications [12]. Thus, the *Warburg effect* does not simply produce a waste product; it establishes a “lactate reservoir” that is intimately sensing the metabolic state of the cell.

### 2.2 Iron addiction and the labile iron pool

Parallel to this glycolytic shift, neoplastic cells exhibit a profound dependency on iron, termed “iron addiction,” to support DNA synthesis and cell cycle progression [12]. Iron is an obligatory cofactor for ribonucleotide reductase, the rate-limiting enzyme in dNTP synthesis, and for the mitochondrial electron transport chain complexes [13]. To satisfy this demand, cancer cells typically orchestrate a dysregulated iron handling program: distinct upregulation of the iron importer TFRC and downregulation of the iron exporter FPN1/SLC40A1 [14]. This import-export imbalance leads to an expansion of the intracellular LIP—a transient, redox-active fraction of iron that is loosely chelated and available for metabolic reactions [15]. While essential for growth, the LIP represents a metabolic liability. Excess Fe^2+^ drives the Fenton reaction, generating highly reactive hydroxyl radicals [16]. In non-malignant cells, this risk is mitigated by storing excess iron in ferritin (FTH1/FTL). However, in cancer cells, the constant degradation of ferritin via ferritinophagy (NCOA4-mediated autophagy) ensures a sustained supply of bioavailable iron [17] (Fig. 1, Center panel). This creates a precarious “iron-rich” state that, while fueling proliferation, places the cell on the precipice of oxidative catastrophe.

### 2.3 The ferroptotic threshold: where lipids meet iron

The convergence of the expanded LIP and metabolic ROS creates the biochemical prerequisites for ferroptosis. This regulated cell death is driven by the iron-dependent peroxidation of PUFAs, particularly arachidonic acid and adrenic acid, found in phospholipids [18]. The sensitivity to ferroptosis is dictated by the composition of the membrane lipidome. Enzymes such as ACSL4 and LPCAT3 are instrumental in enriching cellular membranes with PUFAs, thereby creating the “fuel” for lipid peroxidation [19] (Fig. 1, Right panel). Theoretically, *Warburg*-type tumors should be exquisitely sensitive to ferroptosis. They possess high levels of LIP, high ROS generation from metabolic overdrive, and often heightened lipid synthesis [20]. Yet, clinical observation reveals that many highly glycolytic tumors exhibit intrinsic resistance to ferroptosis inducers [21]. This suggests the existence of a robust, adaptive coupling mechanism that neutralizes this metabolic vulnerability.

### 2.4 Bridging the gap: lactate as the epigenetic adaptor

**Fig. 2:**
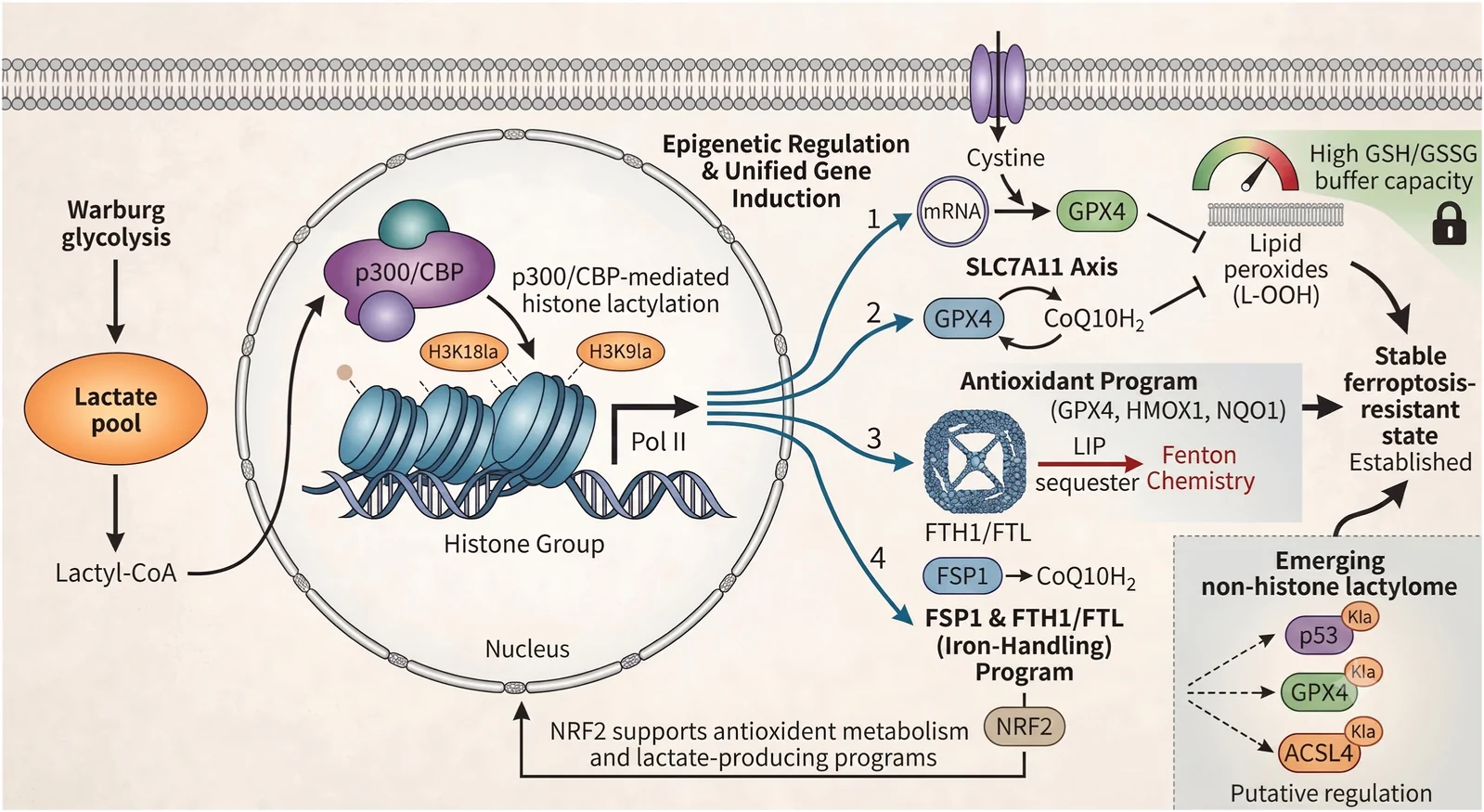
Lactate acts as an epigenetic adaptor to orchestrate a unified ferroptosis-resistant network. In highly glycolytic cancer cells, the Warburg effect drives the accumulation of a substantial intracellular lactate pool, which serves as the primary source for lactyl-CoA synthesis. Within the nucleus, the p300/CBP complex utilizes this lactyl-CoA to mediate histone lactylation, including H3K18la and H3K9la. This epigenetic remodeling facilitates RNA polymerase II-driven transcription of a comprehensive cytoprotective gene signature. The resulting functional anti-ferroptosis defense operates through distinct, parallel branches. Route 1 and Route 2 represent the transcriptional activation of the SLC7A11 and GPX4 axis, which enhances cystine import and glutathione biosynthesis to establish a high buffer capacity that efficiently detoxifies lipid peroxides. Route 3 highlights the upregulation of broader antioxidant programs and iron-handling proteins like FTH1/FTL, which sequester the labile iron pool and prevent ROS generation via Fenton chemistry. Route 4 depicts the activation of FSP1, which regenerates CoQ10H2 to further suppress lipid peroxidation. Concurrently, a feed-forward loop involving NRF2 supports both antioxidant metabolism and sustained lactate production. Finally, an emerging paradigm highlights the putative non-histone lactylation of targets like p53, GPX4, and ACSL4, which may collectively fine-tune this stable ferroptosis-resistant state. Abbreviations: CBP, CREB-binding protein; CoQ10H2, reduced coenzyme Q10; FSP1, ferroptosis suppressor protein 1; FTH1/FTL, ferritin heavy chain/light chain; GPX4, glutathione peroxidase 4; GSH/GSSG, reduced/oxidized glutathione; H3K18la, histone H3 lysine 18 lactylation; H3K9la, histone H3 lysine 9 lactylation; Kla, lysine lactylation; NQO1, NAD(P)H quinone dehydrogenase 1; NRF2, Nuclear Factor Erythroid 2-Related Factor 2; Pol II, RNA polymerase II.

The paradox of “high glycolysis/high iron” but “low ferroptosis” implies that the byproduct of the former actively neutralizes the toxicity of the latter. We propose that this neutralization is not merely chemical but transcriptional [3]. For lactate to exert epigenetic control, it must first be converted into lactyl-CoA, a process potentially mediated by ACSS2 (acyl-CoA synthetase short-chain family member 2) or derived from the mitochondrial oxidation of lactate [3]. This lactyl-CoA serves as the substrate for HATs (histone acetyltransferases), such as p300/CBP (E1A binding protein p300/CREB-binding protein), which have been shown to “moonlight” as histone lactyltransferases [22]. This enzymatic flexibility allows the cell to translate its glycolytic rate into a specific chromatin mark (H3K18la, H3K9la, etc.) [23]. Therefore, the accumulation of lactate in Warburg-phenotype cells acts as a metabolic timer. As glucose flux increases, so does the “lactyl-tone” of the chromatin. We hypothesize that this lactyl-tone is preferentially deposited at the promoters of antioxidant and iron-handling genes, effectively “locking” the cell in a ferroptosis-resistant state despite the volatile presence of the LIP [24] (Fig. 2). This mechanism transforms lactate from a metabolic liability into a strategic asset, reinforcing the cell's antioxidant capacity in direct proportion to its metabolic activity.

## 3 Histone lactylation: epigenetic rewiring of ferroptosis defenders

If lactate accumulation provides the “fuel” for epigenetic remodeling, Kla serves as the “engine” that drives the transcriptional programs necessary for survival. This section dissects the specific chromatin landscapes orchestrated by Kla, focusing on how this modification selectively activates an anti-ferroptotic gene signature that shields the metabolically stressed tumor cell from lipid peroxidation.

### 3.1 The Writer's block: p300/CBP as the metabolic sensor

The translation of intracellular lactate into chromatin modifications requires a precise enzymatic machinery. The canonical histone acetyltransferase p300 and its paralog CBP have been identified as the primary “writers” of histone lactylation [3]. Crucially, the affinity of p300 for lactyl-CoA is distinct from its affinity for acetyl-CoA, allowing the enzyme to sense the metabolic state of the cell. Under conditions of the *Warburg effect*, where the ratio of intracellular lactyl-CoA to acetyl-CoA increases, p300 switches its substrate preference, effectively transitioning from an acetyltransferase to a lactyltransferase [22].

**Fig. 3:**
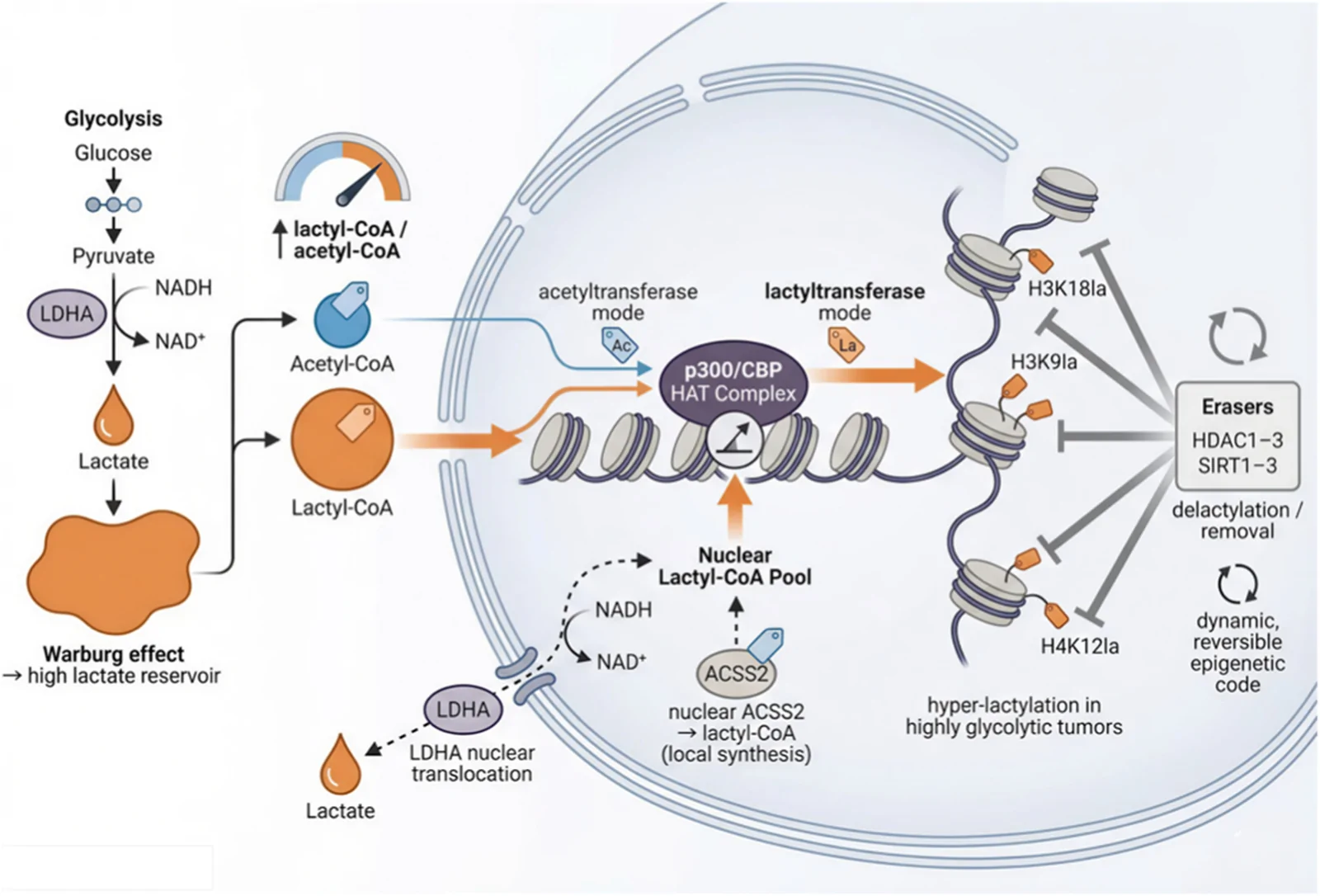
The Warburg effect couples metabolic reprogramming to epigenetic regulation via histone lactylation. *Warburg effect* in cancer cells directs metabolite flux to alter the epigenome. In the cancer cell cytosol, upregulated glycolysis leads to the accumulation of a high lactate reservoir. This metabolic shift significantly increases the intracellular pool of Lactyl-CoA relative to Acetyl-CoA (as indicated by the ratio gauge). Elevated Lactyl-CoA serves as a substrate for nuclear epigenetic modification. Nuclear lactyl-CoA sources include direct influx, nuclear translocation of LDHA, and local synthesis by ACSS2. The p300/CBP HAT complex functions as a metabolic sensor and substrate switch; high levels of Lactyl-CoA promote its activity as a lactyltransferase, leading to the hyper-lactylation of histones at specific marks (e.g., H3K18la, H3K9la, H4K12la). This establishes a dynamic, reversible epigenetic code that is negatively regulated by “eraser” enzymes, including HDAC1–3 and SIRT1–3. Abbreviations: ACSS2, acyl-CoA synthetase short-chain family member 2; CBP, CREB-binding protein; H3K18la, histone H3 lysine 18 lactylation; H3K9la, histone H3 lysine 9 lactylation; H4K12la, histone H4 lysine 12 lactylation; HDAC1–3, histone deacetylases 1–3; LDHA, lactate dehydrogenase A; SIRT1–3, Sirtuins 1–3.

This ‘writer’ activity is fundamentally dependent on the availability of nuclear lactyl-CoA [3]. While initial models posited that this substrate is supplied either by the nuclear translocation of metabolic enzymes like LDHA or via local synthesis by nuclear ACSS2, the broader metabolic community remains actively divided on the latter mechanism [25]. Classically, ACSS2 is an acetate-utilizing enzyme, and its catalytic efficiency and substrate binding affinity for lactate remain mechanistically controversial under physiological intracellular concentrations [26]. To reconcile this biochemical discrepancy, emerging paradigms propose alternative metabolic bypasses for nuclear lactate activation [27,28]. It is highly plausible that other members of the acyl-CoA synthetase family with broader substrate promiscuity (such as ACSS1 or ACSS3), or yet-uncharacterized CoA-transferases, might mediate this activation [29]. Furthermore, alternative models suggest that lactyl-CoA could be generated via a distinct cytosolic or mitochondrial ligase before traversing the nuclear envelope. Acknowledging this ‘ACSS2 controversy’ is critical; identifying the bona fide lactyl-CoA synthetase or the specific metabolic bypass remains one of the most pressing bottlenecks in fully decoding how the Warburg effect drives epigenetic remodeling [29]. Conversely, the removal of these marks (“erasers”) is mediated by Class I histone deacetylases (HDAC1–3) and Sirtuins (SIRT1–3), creating a dynamic, reversible epigenetic code [30]. In highly glycolytic tumors, the equilibrium shifts overwhelmingly toward lactylation, resulting in the hyper-lactylation of specific histone residues, most notably H3K18la, H3K9la, and H4K12la (Fig. 3).

Crucially, it must be emphasized that the correlation between high glycolytic flux, the expansion of the intracellular lactate reservoir, and the resultant epigenetic lactylation tone is not strictly linear. Rather, this paradigm is governed by a highly non-linear kinetic network inextricably linked to intracellular pH homeostasis [27,31]. Accelerated glycolysis inevitably induces significant proton accumulation, necessitating rapid efflux coupled with lactate via monocarboxylate transporters [32]. However, within the inherently acidic TME, the saturation dynamics and thermodynamic limits of these monocarboxylate transporters can impede efflux, leading to intracellular lactate retention. Furthermore, the steady-state lactylation tone is rigorously counterbalanced by the enzymatic kinetics of the ‘erasers’ [33]. The catalytic efficiency and substrate affinity of these delactylases are exquisitely sensitive to fluctuations in the nucleocytoplasmic pH and local NAD+/NADH ratios [34]. Consequently, tumor cells must execute a delicate homeostatic balancing act: they must sustain sufficient nuclear hyperlactylation to drive the anti-ferroptotic transcriptional program, while simultaneously preventing lethal intracellular acidification that would otherwise denature the chromatin architecture and the epigenetic enzymatic machinery itself [35].

### 3.2 Fortifying the glutathione shield: the H3K18la-SLC7A11 axis

The primary defense against ferroptosis is the System $X_{c}^{-}$/GSH/GPX4 axis. System $X_{c}^{-}$ imports cystine for GSH (glutathione) synthesis, which is then used by GPX4 (Glutathione Peroxidase 4) to reduce toxic lipid hydroperoxides into harmless alcohols [36].

Recent evidence suggests a potential regulatory connection between H3K18la and the upregulation of this axis; we hypothesize that H3K18la enrichment at the SLC7A11 promoter serves as a critical epigenetic driver of this antioxidant defense [[37], [38], [39]]. In ocular melanoma and hepatocellular carcinoma models, H3K18la has been shown to enrich at the promoter regions of SLC7A11, driving its transcription even in the absence of canonical NRF2 (Nuclear Factor Erythroid 2-Related Factor 2) activation [23]. We propose that this represents a fundamental “metabolic coupling” mechanism: the more glucose the tumor consumes, the more lactate it produces, which in turn epigenetically enforces higher expression of SLC7A11. This ensures that the high metabolic flux is matched by an equally high capacity for cystine import, effectively neutralizing the ROS byproducts of metabolism before they can trigger ferroptosis.

**Fig. 4:**
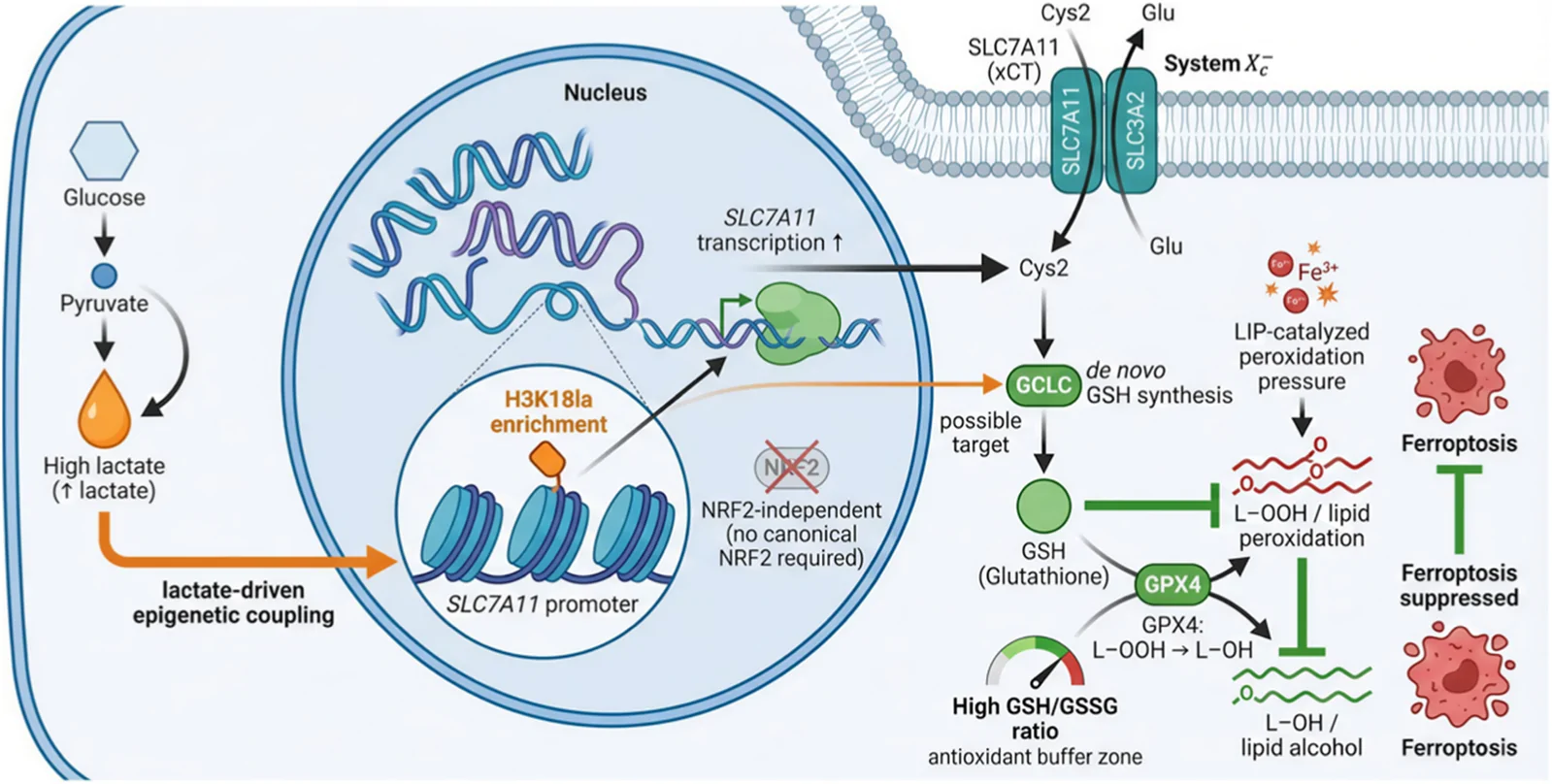
Warburg effect-derived lactate drives an H3K18la-dependent antioxidant axis to suppress ferroptosis. In the cancer cell cytosol, *Warburg* glycolysis leads to a high flux of glucose to pyruvate and subsequent accumulation of a large intracellular lactate reservoir. High lactate load drives epigenetic coupling, resulting in the specific enrichment of histone lactylation mark H3K18la at the SLC7A11 promoter in the nucleus. This H3K18la mark directly activates SLC7A11 transcription via a mechanism that is independent of canonical NRF2 signaling. Upregulated SLC7A11 protein increases membrane System $X_{c}^{-}$ activity, enhancing cystine import. Elevated intracellular cystine fuels de novo GSH synthesis, maintaining a high GSH/GSSG antioxidant buffer zone. GPX4 utilizes this robust GSH pool to resolve toxic L-OOH—generated by LIP-catalyzed pressure—into benign L-OH, thereby preventing lipid peroxidation-induced membrane damage and suppressing ferroptosis. Abbreviations: GCLC, glutamate-cysteine ligase catalytic subunit; GPX4, glutathione peroxidase 4; GSH/GSSG, reduced/oxidized glutathione; H3K18la, histone H3 lysine 18 lactylation; LIP, labile iron pool; L-OOH, lipid hydroperoxides; L-OH, lipid alcohols; NRF2, Nuclear Factor Erythroid 2-Related Factor 2; SLC7A11, solute carrier family 7 member 11.

Furthermore, enzymes involved in de novo GSH synthesis, such as GCLC (Glutamate-Cysteine Ligase Catalytic Subunit), may also be targets of this lactylation-driven program. By maintaining a high GSH/GSSG ratio via epigenetic licensing, the tumor cell creates a “buffer zone” that prevents the LIP-catalyzed lipid peroxidation chain reaction [40] (Fig. 4).

### 3.3 Desaturating the lipidome: Shifting from PUFAs to MUFAs

Ferroptosis requires a specific lipid substrate: PUFAs incorporated into membrane phospholipids. A key determinant of ferroptosis sensitivity is the ratio of PUFAs (pro-ferroptotic) to monounsaturated fatty acids [41].

**Fig. 5:**
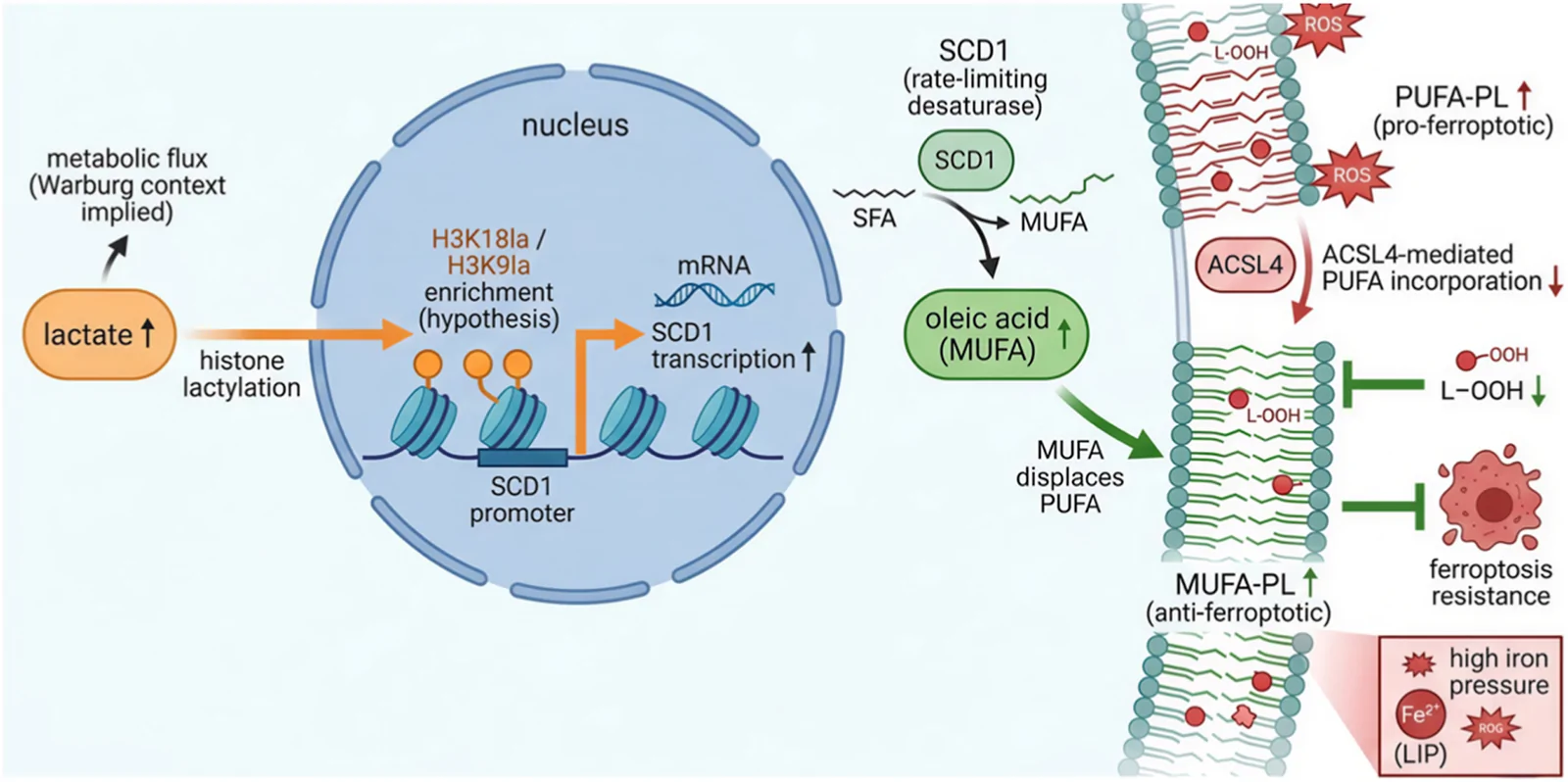
Histone lactylation drives lipidome remodeling via SCD1 to confer ferroptosis resistance. This schematic illustrates how lactate-driven epigenetic rewiring alters membrane lipid composition to evade oxidative damage. Under the *Warburg* metabolic context, increased glycolytic flux elevates the intracellular lactate pool. This leads to the proposed enrichment of histone lactylation marks (H3K18la and H3K9la) at the promoter region of SCD1, driving its mRNA transcription. As the rate-limiting desaturase, upregulated SCD1 actively converts SFA into MUFA, such as oleic acid. These newly synthesized MUFAs subsequently displace pro-ferroptotic PUFA within the plasma membrane, thereby downregulating PUFA-PL incorporation. By depleting the membrane of oxidizable PUFA substrates, the generation of lethal L-OOH is effectively blocked, conferring robust ferroptosis resistance even under conditions of high intracellular iron pressure and ROS accumulation. Abbreviations: ACSL4, Acyl-CoA synthetase long-chain family member 4; H3K18la, Histone H3 lysine 18 lactylation; H3K9la, Histone H3 lysine 9 lactylation; L-OOH, Lipid hydroperoxides; MUFA, Monounsaturated fatty acids; PUFA, Polyunsaturated fatty acids; PUFA-PL, Polyunsaturated fatty acid-phospholipids; ROS, Reactive oxygen species; SCD1, Stearoyl-CoA Desaturase 1; SFA, Saturated fatty acids.

Emerging data points to histone lactylation as a regulator of lipid desaturation enzymes. Specifically, Stearoyl-CoA Desaturase 1 (SCD1), the rate-limiting enzyme converting saturated fatty acids to MUFAs (monunsaturated fatty acids), is often upregulated in aggressive cancers. Based on the established link between lactate and epigenetic remodeling, we propose a model in which H3K18la or H3K9la marks enrich at the SCD1 promoter to drive the synthesis of MUFAs; however, this regulatory axis warrants further validation. These MUFAs displace PUFAs from the plasma membrane, reducing the abundance of oxidizable substrates available for ACSL4-mediated incorporation [42]. Through this “lipidome remodeling,” histone lactylation fundamentally alters the biophysical properties of the membrane, rendering it resistant to oxidative damage even in the presence of high iron levels (Fig. 5).

### 3.4 The NRF2-Lactylation Nexus: A feed-forward loop

**Fig. 6:**
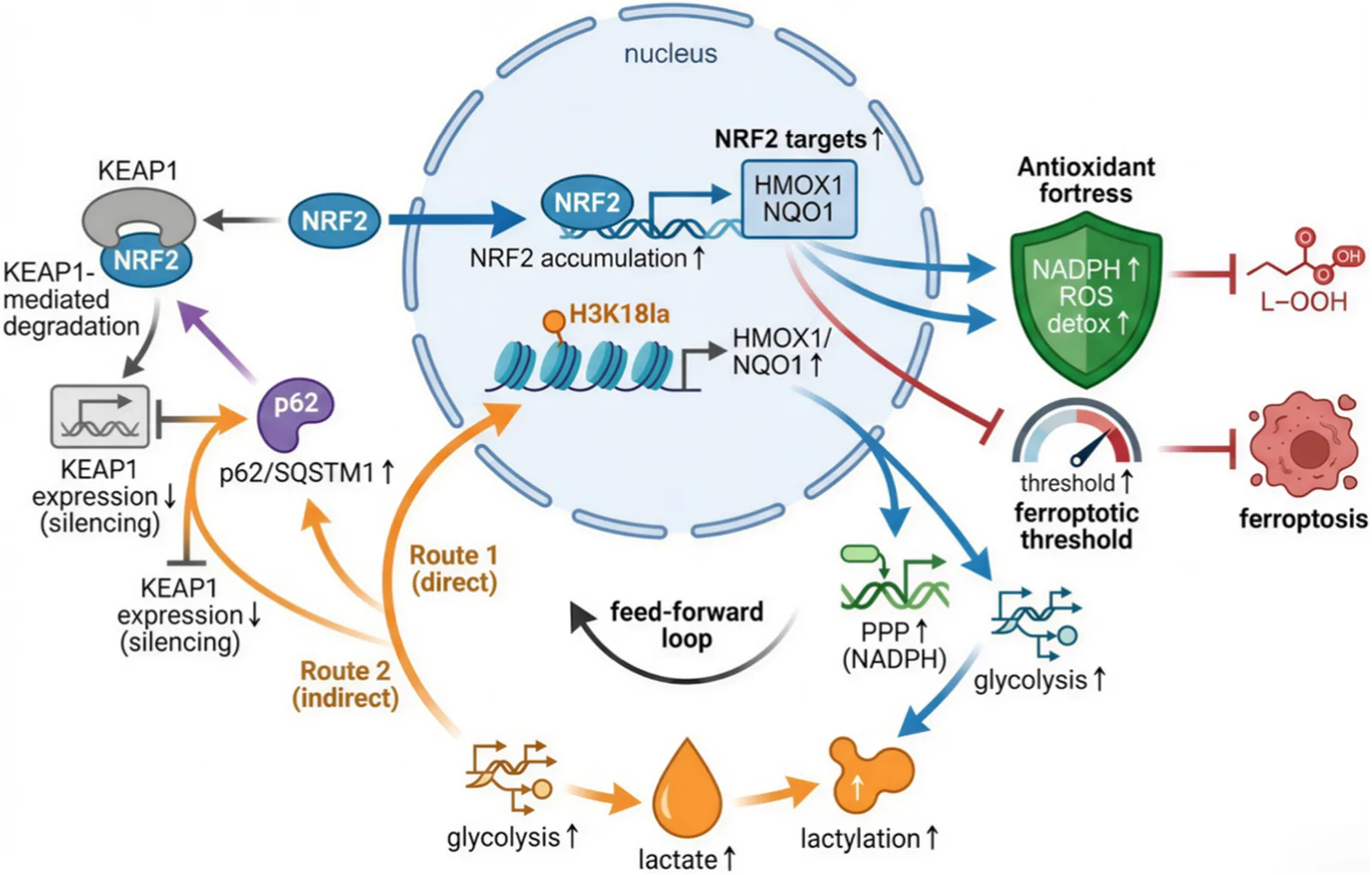
The NRF2–lactylation feed-forward loop establishes an antioxidant fortress to confer ferroptosis resistance. This schematic illustrates a metabolic-epigenetic circuit where lactate metabolism reinforces antioxidant defense. In cancer cells, upregulated glycolysis leads to increased lactate production, which drives overall protein lactylation. Lactylation activates the NRF2 pathway via two distinct mechanisms. Route 1 (direct) shows that histone lactylation, specifically H3K18la, directly promotes the nuclear transcription of NRF2 target genes such as HMOX1 and NQO1. Route 2 (indirect) demonstrates that lactylation leads to the upregulation of p62/SQSTM1 and the silencing of KEAP1 expression. This prevents KEAP1-mediated degradation of NRF2, resulting in NRF2 stabilization and nuclear accumulation. Accumulated nuclear NRF2 drives a transcriptional program that builds an antioxidant fortress characterized by increased NADPH levels and ROS detoxification capacity. This shields the cell from lipid peroxidation, thereby elevating the ferroptotic threshold and preventing ferroptosis. Furthermore, NRF2 signaling upregulates the Pentose Phosphate Pathway and glycolysis, increasing lactate production and reinforcing the lactylation-driven cycle in a sustained feed-forward loop. Abbreviations: Abbreviations: H3K18la, Histone H3 lysine 18 lactylation; HMOX1, Heme oxygenase 1; KEAP1, Kelch-like ECH-associated protein 1; NADPH, Nicotinamide adenine dinucleotide phosphate; NQO1, NAD(P)H quinone dehydrogenase 1; NRF2, Nuclear Factor Erythroid 2-Related Factor 2; p62/SQSTM1, Sequestosome-1; PPP, Pentose phosphate pathway; ROS, Reactive oxygen species.

NRF2 is the master transcriptional regulator of the antioxidant response and a known suppressor of ferroptosis [43]. While NRF2 is typically regulated by KEAP1-mediated degradation, epigenetic mechanisms play a crucial role in its sustained activation in cancer. Lactylation may intersect with the NRF2 pathway at two levels. First, H3K18la can directly activate the transcription of *NFE2L2* or its downstream targets, acting synergistically with oxidative stress signals (Fig. 6, Route 1). Second, and perhaps more insidiously, lactate-derived lactylation may silence *KEAP1* expression or promote the expression of p62/SQSTM1, leading to constitutive NRF2 accumulation (Fig. 6*, Route 2)* [44]. This establishes a “feed-forward loop”: NRF2 promotes metabolic reprogramming toward the pentose phosphate pathway and glycolysis, while lactate-mediated epigenetics reinforces NRF2 activity. This axis is proposed to establish a ‘feed-forward loop’ that creates an antioxidant fortress, although the kinetic interplay of these events requires further investigation.

### 3.5 Beyond the nucleus: non-histone lactylation targets

While histone modifications are the best-characterized aspect of lactylation, the post-translational modification of non-histone proteins represents a burgeoning frontier. Just as acetylation regulates the function of cytosolic proteins, it is highly probable that lactylation directly modulates the stability or activity of ferroptosis executioners. For instance, p53 is a known transcriptional repressor of SLC7A11, thereby promoting ferroptosis [45]. If p53 were to undergo lactylation, this modification might inhibit its DNA binding capacity or promote its nuclear export, thereby lifting the repression on SLC7A11. Similarly, the direct lactylation of GPX4 or ACSL4 could alter their enzymatic efficiency or protein stability [46,47]. Although direct evidence for specific non-histone targets in the ferroptosis pathway is currently nascent, the structural similarity between acetyl-CoA and lactyl-CoA suggests that the entire “acetylome” involved in cell death regulation may have a parallel “lactylome” counter-part that warrants urgent investigation [48]. To maintain scientific rigor and guide future investigations, a definitive boundary must be established between empirically validated lactylation targets and those derived via theoretical extrapolation from canonical acylation pathways [3,49]. While the histone lactylation of targets such as SLC7A11 and Arg1, as well as the non-histone lactylation of METTL3, have been robustly validated in specific in vitro and in vivo oncological models, the modulation of key ferroptotic executioners—namely p53, GPX4, ACSL4, and the lipid desaturase SCD1—remains strictly hypothetical. Table 1 systematically delineates the current landscape of these epigenetic and epitranscriptomic targets, explicitly differentiating mechanistic paradigms substantiated by direct experimental evidence from those posited as prospective theoretical models [25,50].

**Table 1:** Delineation of empirically validated versus hypothetical lactylation (Kla) targets in ferroptosis and tumor immunity.

Target Molecule	Modification Type	Putative Biological Consequence	Current Evidence Status	Reference
**SLC7A11**	Histone (H3K18la)	Transcriptional upregulation; enhanced cystine import and GSH synthesis	Experimentally Validated	[1]
**Arg1**	Histone (H3K18la)	Transcriptional activation; induction of M2-like immunosuppressive TAM phenotype	Experimentally Validated	[2]
**METTL3**	Non-histone (K281la)	Enhanced m6A modification; hyperactivation of JAK/STAT3 signaling	Experimentally Validated	[3]
**SCD1**	Histone (H3K18la/H3K9la)	Transcriptional upregulation; MUFA accumulation and PUFA displacement	Strictly Hypothetical	N/A
**p53**	Non-histone (Kla)	Altered DNA-binding affinity or nuclear export; abrogation of SLC7A11 repression	Strictly Hypothetical	N/A
**GPX4**	Non-histone (Kla)	Modulation of enzymatic efficiency or protein stability; augmented lipid peroxide clearance	Strictly Hypothetical	N/A
**ACSL4**	Non-histone (Kla)	Altered catalytic activity; modulation of PUFA-phospholipid membrane enrichment	Strictly Hypothetical	N/A

***Abbreviations:*** ACSL4, Acyl-CoA synthetase long-chain family member 4; Arg1, Arginase-1; CBP, CREB-binding protein; GPX4, Glutathione peroxidase 4; GSH, Glutathione; H3K18la, Histone H3 lysine 18 lactylation; H3K9la, Histone H3 lysine 9 lactylation; JAK, Janus kinase; Kla, Lysine lactylation; m6A, N6-methyladenosine; METTL3, Methyltransferase-like 3; MUFA, Monounsaturated fatty acids; p53, Tumor protein p53; PUFA, Polyunsaturated fatty acids; SCD1, Stearoyl-CoA desaturase 1; SLC7A11, Solute carrier family 7 member 11; STAT3, Signal transducer and activator of transcription 3; TAM, Tumor-associated macrophage.

**Fig. 7:**
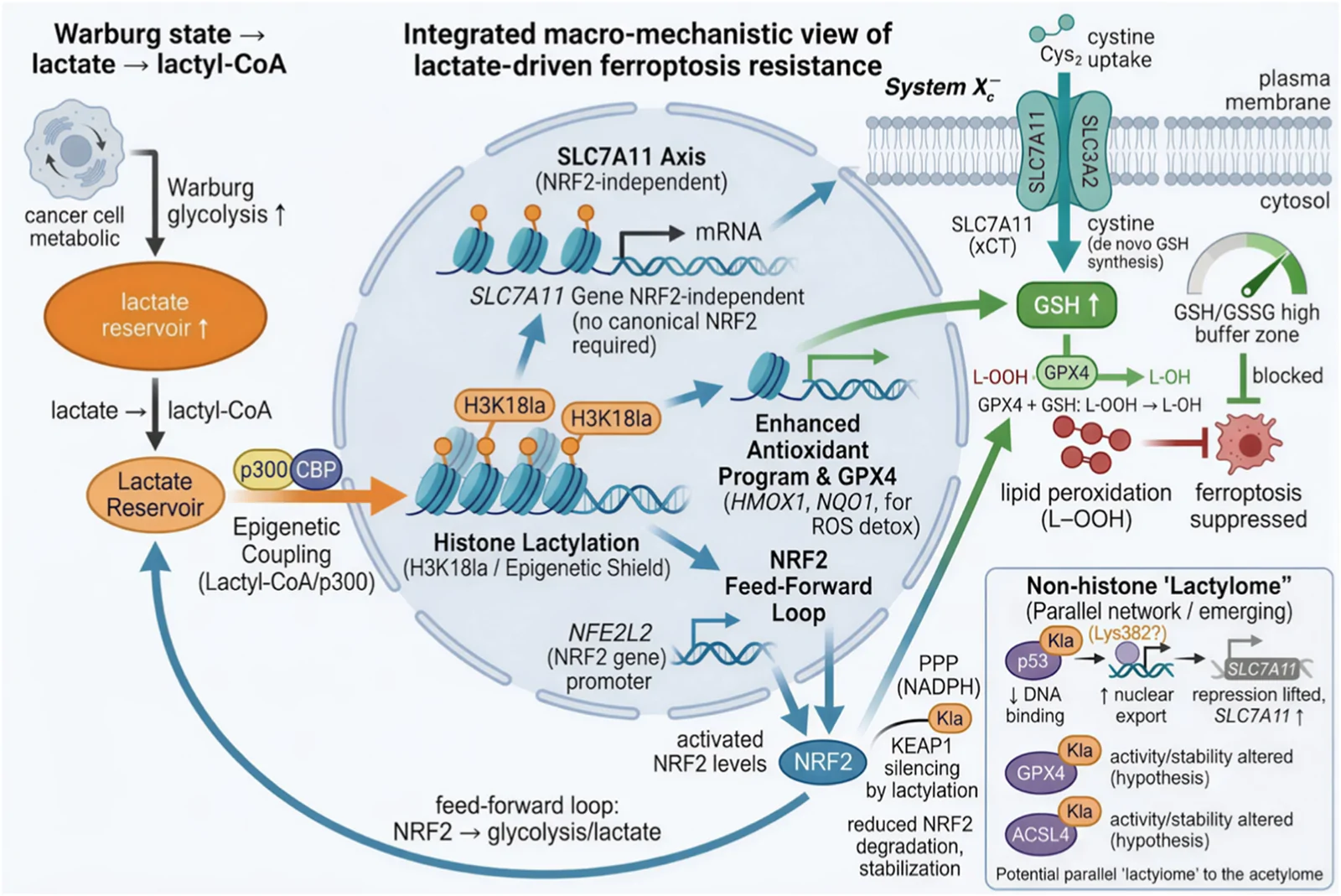
*Warburg* effect-derived lactate orchestrates ferroptosis resistance via unified epigenetic and epitranscriptomic rewiring. This consolidated schematic illustrates the multi-layered defense activated by high glycolytic flux, linking Warburg effect-derived lactate production to profound ferroptosis resistance. (A) The Epigenetic Shield: H3K18la-SLC7A11/GPX4 Axis. Elevated Warburg glycolysis leads to a robust intracellular lactate reservoir, which is converted to lactyl-CoA. The p300/CBP complex utilizes this lactyl-CoA to deposit H3K18la (histone lactylation) marks at specific gene promoter regions (indicated by the large orange coupling arrow to the central nucleosome hub). This deposition acts as a central control, branching into multiple downstream pathways. One major branch (top-right) points directly to the SLC7A11 gene promoter, driving its transcription in an NRF2-independent manner. The enhanced transcription leads to increased System $X_{c}^{-}$ transporter activity (SLC7A11/SLC3A2) at the plasma membrane, facilitating cystine (Cys_2_) uptake and GSH synthesis. Simultaneously, another central branch (middle-right) promotes transcription of GPX4 and other ROS detoxifying genes (e.g., HMOX1, NQO1). These elements collectively buffer LIP-driven lipid peroxidation (L-OOH), suppressing ferroptosis. Prominent gauges show the resulting high antioxidant buffering and low lipid peroxidation. (B) The NRF2 Feed-Forward Loop. Parallel to the direct gene transcription axis, H3K18la drives the transcription of NFE2L2 (the NRF2 gene). Concurrently, a distinct cytosolic pathway (bottom-right) shows that Kl) stabilizes and activates NRF2, partially through KEAP1 silencing, leading to reduced NRF2 degradation. Active NRF2 further consolidates the antioxidant fortress and reinforces lactate production via upregulated PPP (NADPH) and glycolysis, creating a feed-forward loop (indicated by the bottom-left blue arrow back to the lactate reservoir). (C) Non-histone “Lactylome”. A parallel, emerging regulatory network (enclosed box, bottom-right) is highlighted, where Kla modulates key ferroptosis executioners. Kla of p53 leads to reduced DNA binding and increased nuclear export, lifting repression on SLC7A11. The potential (hypothetical) Kla of GPX4 and ACSL4 is suggested to alter their activity and stability. Collectively, these multifaceted mechanisms elevate the overall ferroptotic threshold, creating a comprehensive resistance phenotype under Warburg conditions. Abbreviations: ACSL4, Acyl-CoA synthetase long-chain family member 4; CBP, CREB-binding protein; GPX4, Glutathione peroxidase 4; GSH, Glutathione; H3K18la, Histone H3 lysine 18 lactylation; HMOX1, Heme oxygenase 1; KEAP1, Kelch-like ECH-associated protein 1; Kla, Lysine lactylation; L-OOH, Lipid hydroperoxides; LIP, Labile iron pool; NADPH, Nicotinamide adenine dinucleotide phosphate; NFE2L2, Nuclear factor erythroid 2-related factor 2 gene; NQO1, NAD(P)H quinone dehydrogenase 1; NRF2, Nuclear factor erythroid 2-related factor 2; PPP, Pentose phosphate pathway; SLC3A2, Solute carrier family 3 member 2; SLC7A11, Solute carrier family 7 member 11.

In summary, histone lactylation functions as a master epigenetic switch. By sensing the Warburg state, it orchestrates a multi-layered defense strategy—boosting glutathione synthesis, remodeling the lipidome, and sustaining NRF2 activity—that systematically dismantles the machinery required for ferroptosis (Fig. 7).

## 4 The TME dimension: lactylation orchestrates an anti-ferroptotic immune niche

The “Lactate–Kla–Ferroptosis” axis extends its influence far beyond the neoplastic cell itself, functioning as a potent paracrine mechanism that fundamentally reshapes the tumor immune microenvironment). In the hypoxic, nutrient-deprived core of solid tumors, lactate secreted by glycolytic cancer cells acts as an epigenetic morphogen, diffusing into the stroma to impose a specific lactylation signature on infiltrating immune cells. This metabolic crosstalk effectively dismantles the surveillance machinery required to trigger ferroptosis, transforming the microenvironment into an immunosuppressive niche that buffers the tumor against oxidative stress.

The most well-characterized target of this remodeling is the tumor-associated macrophage (TAM) population. While TAMs exhibit significant plasticity, the lactate-rich environment drives them inexorably toward a pro-tumorigenic, anti-inflammatory M2-like phenotype. A seminal study by *Zhang* et al. established that this polarization is not merely a transient response to cytokines, but a stable epigenetic reprogramming event driven by histone lactylation [31]. Exogenous lactate enters macrophages via MCT1 and serves as a substrate for p300 to deposit H3K18la marks at the promoters of canonical M2 signature genes, most notably Arg*1* (Arginase-1). This lactylation-locked M2 state protects tumor cells from ferroptosis through multiple mechanisms. The upregulation of Arg*1* depletes the microenvironment of l-arginine, a nutrient essential for T cell proliferation, thereby blunting the immune response [51]. Concurrently, rather than acting as ‘iron sinks', M2-like TAMs classically adopt an ‘iron-donor’ phenotype to support the immense metabolic demands of tumor proliferation. This is achieved by upregulating the scavenger receptor CD163 to internalize extracellular haptoglobin-hemoglobin complexes, coupled with the robust efflux of intracellular iron into the TME via the iron exporter FPN1 [52]. While this FPN1-mediated iron feeding fuels rapid tumor growth, it paradoxically expands the LIP within adjacent malignant cells, theoretically pushing them closer to an oxidative catastrophe [53]. To safely harness this iron influx without triggering ferroptosis, the tumor cells are forced into a strict dependency on the lactylation-driven antioxidant shield. Furthermore, to actively buffer the oxidative risk associated with this iron feeding, these reprogrammed TAMs concurrently secrete paracrine survival factors, such as TGF-β. These factors promote epithelial-to-mesenchymal transition states that are characterized by heightened glutathione synthesis, thereby providing an additional layer of robust ferroptosis resistance [51]. Furthermore, these macrophages secrete cytokines such as TGF-β, which can promote epithelial-to-mesenchymal transition states that are paradoxically resistant to oxidative stress via enhanced glutathione synthesis.

Beyond the myeloid lineage, the lactate-reprogrammed TME systematically disrupts the “immunogenic ferroptosis” axis. An effective anti-tumor response relies on activated cytotoxic T lymphocytes releasing IFN-γ (Interferon-gamma), which binds to tumor cells and transcriptionally downregulates SLC7A11, effectively starving them of cysteine and sensitizing them to lipid peroxidation [54]. However, high concentrations of lactate have been shown to induce mitochondrial dysfunction and stem-like exhaustion in CD8^+^ T cells. It is hypothesized that histone lactylation plays a direct role in this dysfunction by enforcing a closed chromatin conformation at effector loci or promoting the expression of exhaustion markers like PD-1. By suppressing the sustained production of IFN-γ, the tumor ensures that SLC7A11 expression remains uninhibited, effectively severing the immune-ferroptosis connection. Consequently, even if the adaptive immune system recognizes the tumor, the metabolic brake imposed by lactylation prevents T cells from delivering the lethal oxidative blow.

**Fig. 8:**
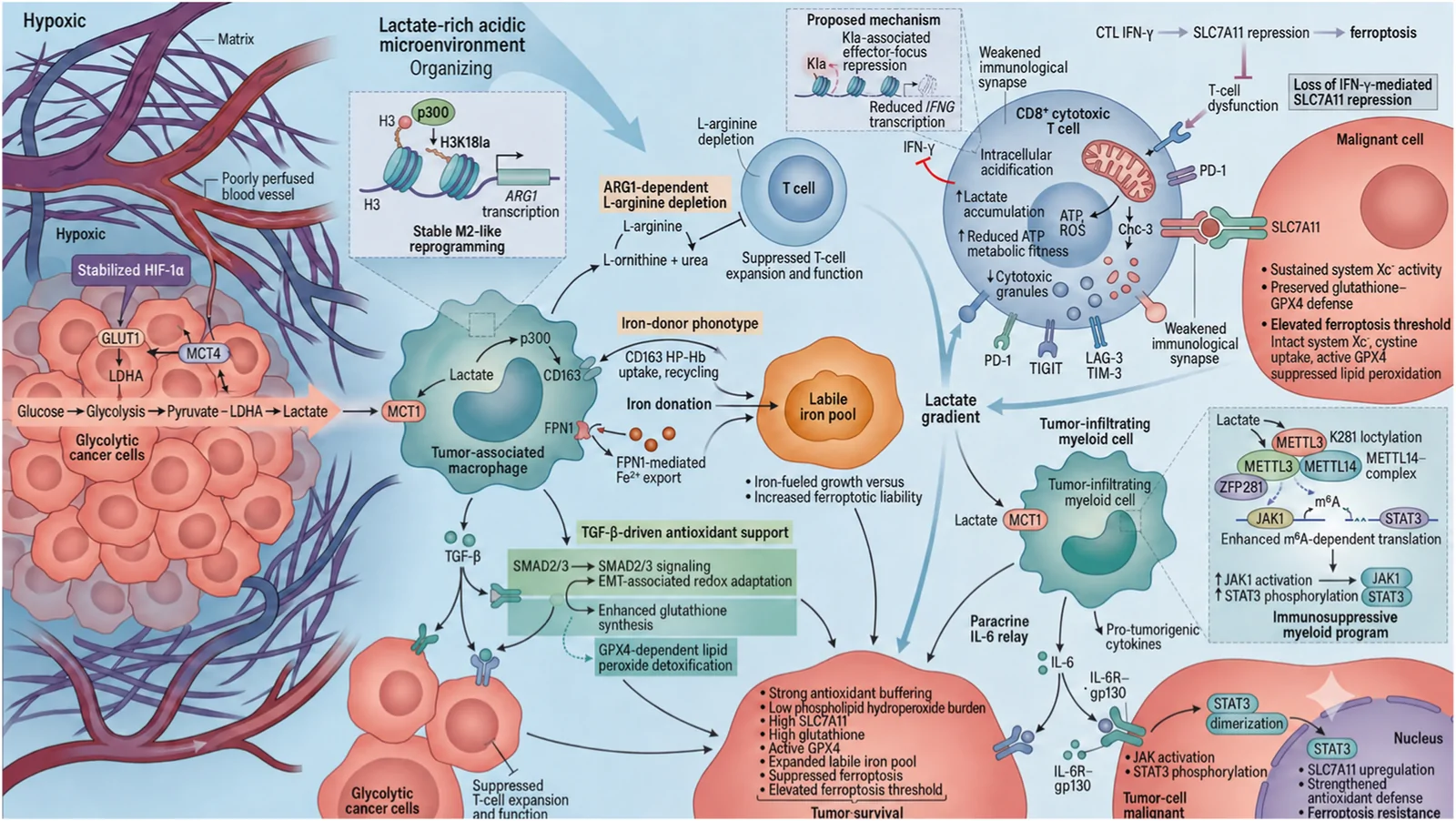
Lactylation orchestrates an anti-ferroptotic immune niche in the TME. Within hypoxic and poorly perfused tumor regions, glycolytic cancer cells exhibit a Warburg-like metabolic phenotype characterized by increased glucose uptake, LDHA-dependent lactate production and MCT4-mediated export of lactate and H^+^. The resulting lactate-rich acidic microenvironment functions as a paracrine metabolic field that reprograms tumor-infiltrating immune cells and disrupts immune-mediated ferroptosis. Lactate is imported into TAMs through MCT1 and promotes p300-dependent deposition of H3K18la at the ARG1 locus, thereby stabilizing an M2-like immunoregulatory phenotype. Increased ARG1 activity depletes extracellular l-arginine and generates l-ornithine and urea, restricting T-cell proliferation and effector function. In parallel, lactate-reprogrammed TAMs acquire an iron-donor phenotype through increased CD163-mediated uptake and recycling of haptoglobin–haemoglobin complexes and FPN1-dependent Fe^2+^ export. Iron transfer to adjacent malignant cells expands the labile iron pool and supports tumor growth, but simultaneously increases ferroptotic liability, creating a strict requirement for reinforced antioxidant protection. TAM-derived TGF-β further promotes EMT (epithelial-to-mesenchymal transition)-associated redox adaptation, enhanced glutathione synthesis and GPX4-dependent detoxification of phospholipid hydroperoxides, allowing tumor cells to tolerate increased iron availability without undergoing ferroptosis. High extracellular lactate also impairs CD8^+^ cytotoxic T-cell function by inducing intracellular acidification, mitochondrial dysfunction, reduced ATP production, increased mitochondrial ROS and progressive exhaustion associated with inhibitory receptors such as PD-1, TIGIT, LAG-3 and TIM-3. A proposed, context-dependent mechanism further involves Kla-associated chromatin restriction at effector loci, including IFNG, thereby limiting sustained IFN-γ production. Because CTL-derived IFN-γ normally suppresses tumor-cell SLC7A11 expression and sensitizes malignant cells to cystine deprivation and lipid peroxidation, lactate-driven T-cell dysfunction preserves system X_c_^−^ activity, glutathione synthesis and GPX4 function, effectively severing the immune–ferroptosis connection. In tumor-infiltrating myeloid cells, intracellular lactate additionally induces METTL3 lactylation at lysine 281. Lactylated METTL3, together with METTL14 and ZFP281, enhances m6A-dependent expression of JAK1 and STAT3, amplifying intrinsic JAK–STAT3 signaling and consolidating an immunosuppressive myeloid program. STAT3-activated myeloid cells subsequently release IL-6 and other pro-tumorigenic cytokines, which act through an intercellular paracrine relay on IL-6R–gp130 expressed by neighboring cancer cells. Secondary activation of tumor-cell STAT3 increases SLC7A11 transcription and strengthens the glutathione–GPX4 antioxidant axis. Collectively, lactate-dependent metabolic, epigenetic and epitranscriptomic reprogramming suppresses antitumor immunity, buffers oxidative stress, elevates the ferroptotic threshold and establishes a tumor-supportive microenvironment resistant to immune-mediated ferroptotic cell death. Abbreviations: ARG1, arginase 1; CTL, cytotoxic T lymphocyte; EMT, epithelial-to-mesenchymal transition; FPN1, ferroportin 1; GPX4, glutathione peroxidase 4; H3K18la, histone H3 lysine 18 lactylation; Kla, lysine lactylation; LDHA, lactate dehydrogenase A; MCT1/4, monocarboxylate transporter 1/4; m6A, N6-methyladenosine; TAM, tumor-associated macrophage; TME, tumor microenvironment.

Adding another layer of complexity is the intersection of lactylation with epitranscriptomics. Recent evidence reveals that intracellular lactate promotes the lactylation of METTL3, a key RNA m6A methyltransferase, in tumor-infiltrating myeloid cells [55]. Specifically, lactylation of METTL3 at Lysine 281 enhances its interaction with ZFP281, leading to increased m6A modification and translation of JAK1 and STAT3 mRNAs [50]. Crucially, this ‘epitranscriptomic-epigenetic’ axis operates via a distinct intercellular paracrine relay rather than a direct intracellular modulation of tumor transcripts [29,50]. The hyper-activation of the JAK/STAT3 signaling pathway occurs intrinsically within the myeloid cells, consolidating their immunosuppressive profile. Subsequently, these STAT3-activated myeloid cells actively secrete an array of paracrine survival factors, such as IL-6 and other pro-tumorigenic cytokines, into the TME [56]. These soluble mediators engage their respective cognate receptors on the surface of adjacent malignant cells, triggering a secondary downstream cascade that activates the tumor cells' intrinsic STAT3 [57]. Given that STAT3 is a canonical transcriptional activator of SLC7A11, this cytokine-mediated intercellular communication ultimately fortifies the tumor cell's systemic antioxidant capacity [58]. Thus, myeloid METTL3 lactylation orchestrates ferroptosis evasion indirectly, acting as an upstream amplifier of tumor-protective paracrine signaling. Thus, lactylation acts as the central architect of a ferroptosis-resistant ecosystem, recruiting and rewiring the immune stroma to serve as accomplices that shield the malignancy from both immune attack and oxidative collapse (Fig. 8).

### 4.1 Therapeutic implications: dismantling the epigenetic shield to unleash ferroptosis

The delineation of the “Lactate–Kla–Ferroptosis” axis unveils a new paradigm for therapeutic intervention. Historically, the clinical translation of ferroptosis inducers has been hindered by the rapid emergence of adaptive resistance mechanisms, particularly the upregulation of antioxidant systems that buffer lipid peroxidation. We posit that these resistance mechanisms are not intrinsic to the cancer cell's lineage but are actively maintained by the metabolic-epigenetic coupling described herein. Therefore, a strategy of “synthetic lethality"—targeting the lactylation machinery to dismantle the antioxidant shield before administering a ferroptotic trigger—represents a promising avenue to overcome the therapeutic stalemate in refractory solid tumors.

**Fig. 9:**
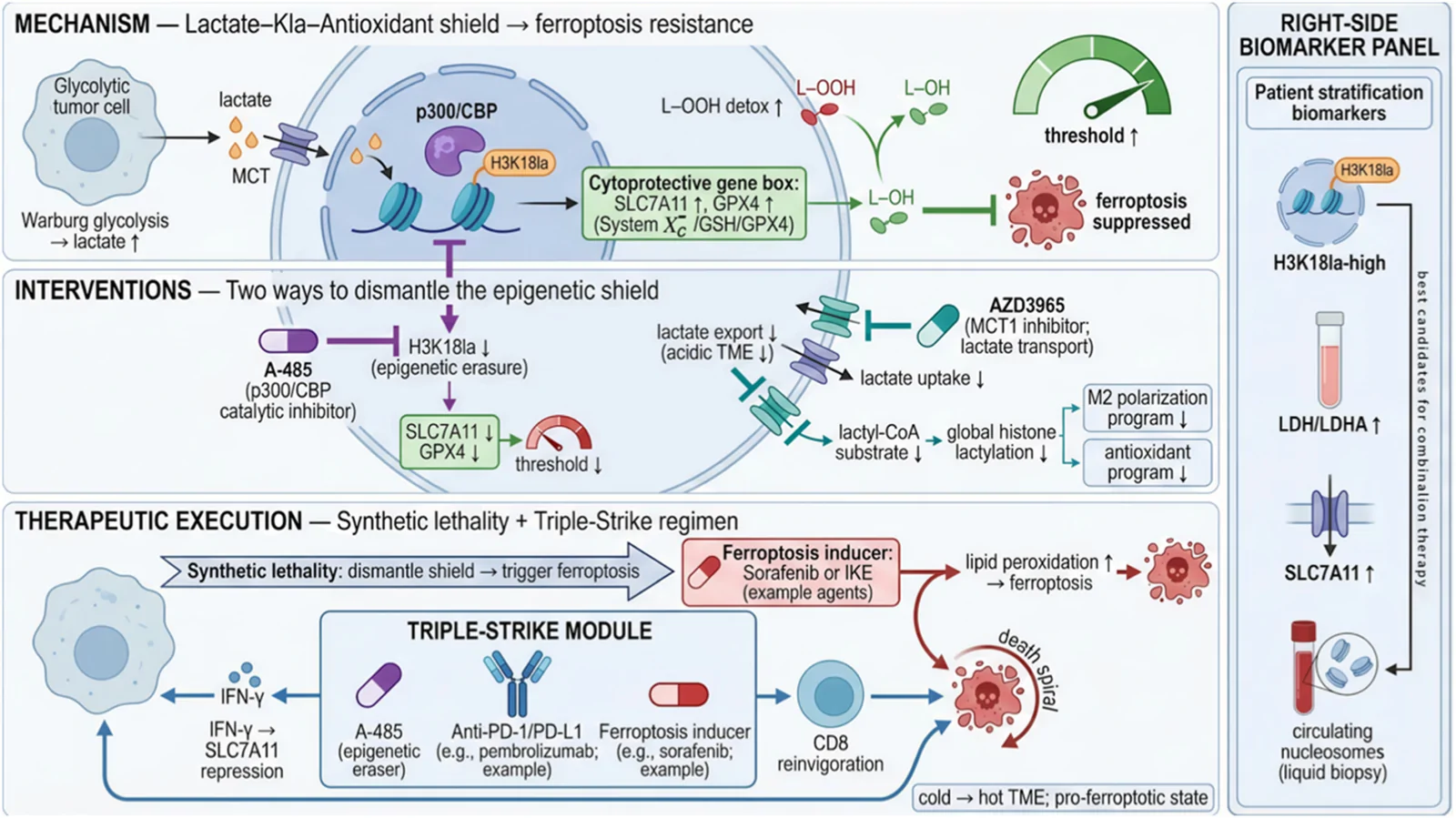
Targeting the lactate–epigenetic–ferroptosis axis for combinatorial cancer therapy. This schematic illustrates the mechanism of lactate-driven ferroptosis resistance and a proposed multi-modal therapeutic strategy to overcome it. **(Top Lane)** Mechanism: In glycolytic tumor cells, the Warburg effect fuels high lactate production. Lactate is transported into the nucleus and utilized as a substrate by the p300/CBP complex to establish histone lactylation marks, specifically H3K18la. This epigenetic modification activates a cytoprotective gene program (upregulating SLC7A11 and GPX4), which enhances the detoxification of lipid hydroperoxides (L-OOH) into benign alcohols (L-OH). This antioxidant shield raises the ferroptotic threshold, suppressing ferroptotic cell death. (Middle Lane) Interventions: Two strategies are shown to dismantle this epigenetic shield. **(Left)** A-485, a p300/CBP catalytic inhibitor, blocks H3K18la writing, leading to epigenetic erasure, downregulation of SLC7A11/GPX4, and a lowered ferroptotic threshold. **(Right)** AZD3965, an MCT1 inhibitor, disrupts lactate transport across the membrane. This leads to intracellular acidification and reduces the nuclear lactyl-CoA substrate pool, globally suppressing lactylation-driven antioxidant and M2 polarization programs. (Bottom Lane) Therapeutic Execution: A synthetic lethality approach involves first dismantling the protective shield (via A-485 or AZD3965), rendering cells vulnerable to subsequent treatment with a ferroptosis inducer (e.g., sorafenib or IKE) to trigger toxic lipid peroxidation. The “Triple-Strike Module” combines the epigenetic eraser (A-485), immune checkpoint blockade (anti-PD-1/PD-L1), and a ferroptosis inducer. In this regimen, ICB reinvigorates CD8^+^ T cells, promoting IFN-γ secretion which further represses tumor cell SLC7A11. This synergistic attack drives a pro-ferroptotic “death spiral,” converting an immunosuppressive (“cold”) TME into an inflamed (“hot”) state. **(Right Panel)** Biomarkers: Patient stratification biomarkers used to identify candidates best suited for this combination therapy include high levels of tumor H3K18la, LDH/LDHA, SLC7A11, or detectable circulating nucleosomes. Abbreviations: CBP, CREB-binding protein; GSH, glutathione; H3K18la, histone H3 lysine 18 lactylation; ICB, immune checkpoint blockade; IFN-γ, Interferon-gamma; IKE, Imidazole ketone erastin; Kla, lysine lactylation; LDH/LDHA, lactate dehydrogenase A; L-OOH, lipid hydroperoxides; L-OH, lipid alcohols; MCT1, monocarboxylate transporter 1; SLC7A11, solute carrier family 7 member 11; TME, tumor microenvironment.

The most direct approach to sever this link is to target the enzymatic “writers” of the lactyl-code. The histone acetyltransferase p300/CBP has been identified as the primary enzyme responsible for catalyzing H3K18la modification. Consequently, the use of selective catalytic inhibitors of p300/CBP, such as A-485, offers a precision oncology tool [59]. Unlike broad-spectrum HDAC inhibitors, A-485 specifically abrogates the deposition of H3K18la marks at the promoters of cytoprotective genes. Preclinical models indicate that A-485 treatment rapidly erases the “lactyl-tone” of the chromatin, leading to the transcriptional collapse of the SLC7A11 and GPX4 axes (Fig. 9, Middle Lane) [60]. This ‘epigenetic erasure’ effectively lowers the ferroptotic threshold, priming previously resistant, highly glycolytic tumors for targeted ferroptotic execution [61,62]. In this context, we highlight sorafenib as a preferred therapeutic agent. Originally developed as a multi-kinase inhibitor, sorafenib has been robustly validated as a potent ferroptosis inducer [63,64]. Mechanistically, sorafenib induces ferroptosis through a dual-pronged blockade of the cystine/glutamate antiporter: it directly and competitively inhibits the functional activity of System $X_{c}^{-}$, and simultaneously represses the expression of the SLC7A11 subunit by promoting the proteasomal degradation of c-Myc [65,66]. Crucially, as an FDA-approved drug with a well-characterized safety profile and established dosing regimens for solid tumors, sorafenib presents an immediate, highly translatable candidate for clinical repurposing in combinatorial regimens [64].

However, other ferroptosis-inducing compounds could also be integrated into this proposed therapeutic strategy. Imidazole ketone erastin (IKE), an optimized derivative of the classical FIN erastin, serves as an excellent alternative due to its superior solubility and in vivo pharmacokinetic profile for System $X_{c}^{-}$ inhibition [67]. Furthermore, direct GPX4 inhibitors, such as RSL3 or ML162, offer an alternative pharmacological route by bypassing the upstream amino acid transport and directly inactivating the lipid peroxide scavenging enzyme [64,68]. While these direct GPX4 inhibitors are highly potent in preclinical models, their current utility is largely restricted to tool compounds due to suboptimal in vivo bioavailability and potential systemic toxicity. Additionally, FDA-approved drugs like sulfasalazine exhibit mild System $X_{c}^{-}$ inhibitory effects and could theoretically be repurposed, though their potency is significantly lower than that of sorafenib [69]. Therefore, while multiple FIN classes exist, the combination of epigenetic erasers with clinically mature agents like sorafenib currently offers the most viable path to clinical translation. However, the clinical translation of broad-spectrum p300/CBP-targeted epigenetic inhibitors such as A-485 presents formidable translational hurdles [70,71]. As the p300/CBP complex acts as a master transcriptional co-activator indispensable for basal transcription across nearly all healthy somatic cell lineages, sustained systemic inhibition carries a high risk of globally disrupting histone modification landscapes, including canonical acetylation marks and Kla [71,72]. Such pervasive epigenetic dysregulation would precipitate severe, dose-limiting systemic toxicities, substantially narrowing the therapeutic window of these agents [71]. Accordingly, unlocking the full clinical potential of this synthetic lethality strategy requires refined translational approaches to mitigate off-target effects and enhance tumor selectivity.

One promising strategy is the adoption of intermittent pulse-dosing regimens, which transiently dismantle the tumor's epigenetic protective program to create a defined temporal window for ferroptosis induction, while granting healthy tissues sufficient recovery time to restore basal transcriptional homeostasis [[73], [74], [75]]. Alternatively, integrating tumor-targeted nanomedicine delivery platforms—such as pH-responsive liposomes or ligand-conjugated nanoparticles—can restrict p300/CBP inhibition selectively to the acidic, lactate-enriched TME, thereby maximizing antitumor efficacy while preserving systemic physiological homeostasis [76].

Parallel to targeting the writer, disrupting the metabolic supply chain provides a complementary strategy. The pharmacological inhibition of MCT1/MCT4 with agents like AZD3965 not only blocks the export of lactate—thereby neutralizing the acidic immunosuppressive microenvironment—but also inhibits the uptake of lactate by oxidative tumor cells and stromal macrophages. By starving the intracellular pool of lactate, these inhibitors deprive p300 of the lactyl-CoA substrate required for histone modification. This substrate depletion leads to a global reduction in histone lactylation, dismantling the transcriptional programs that support M2 macrophage polarization and intrinsic antioxidant defense. Clinical trials evaluating MCT1 inhibitors are currently underway, and their combination with ferroptosis inducers represents a logical next step to exploit the metabolic vulnerability of glycolytic tumors.

Perhaps the most compelling translational horizon lies in the integration of these metabolic-epigenetic interventions with ICB (immune checkpoint blockade). Current ICB therapies, such as anti-PD-1/PD-L1 antibodies, often fail in “cold” tumors where the microenvironment is dominated by suppressive myeloid cells and exhausted T cells. As discussed, lactylation is a key driver of this immunosuppressive landscape. Therefore, a “Triple-Strike” regimen can be envisioned (1) An epigenetic eraser (e.g., A-485): to strip the tumor of its antioxidant defenses and prevent M2 polarization (2) An immune checkpoint inhibitor (e.g., Pembrolizumab); to reinvigorate CD8^+^ T cells; and (3) A ferroptosis inducer (e.g., Sorafenib) to provide the lethal oxidative trigger. In this scenario, the inhibition of lactylation acts as a “chemo-immunogen,” converting the TME from a cold, pro-survival state to a hot, pro-ferroptotic state (Fig. 9, Bottom Lane). This synergy would allow the adaptive immune system to amplify the effects of the ferroptosis inducer, utilizing T cell-derived IFN-γ to permanently repress System $X_{c}^{-}$ and lock the tumor in a death spiral.

Finally, the successful clinical implementation of these strategies necessitates the development of robust biomarkers for patient stratification. Not all tumors will be dependent on the lactylation axis; those with low glycolytic rates or alternative antioxidant mechanisms may not respond. We propose that the quantification of H3K18la levels in tumor biopsies via immunohistochemistry, or potentially in circulating nucleosomes, could serve as a predictive biomarker for “lactylation addiction.” However, the clinical translation of H3K18la liquid biopsies faces substantial technical hurdles that must be acknowledged [77]. Circulating plasma inherently contains massive metabolic background noise, including generic nucleosomes released by normal apoptotic immune cells and lactate dehydrogenase liberated from incidental erythrocyte lysis [78,79]. Distinguishing a bona fide, tumor-specific H3K18la signal from this systemic baseline represents a formidable analytical challenge. Overcoming this barrier will mandate the development of ultra-sensitive and highly specific detection platforms [80]. Future diagnostic efforts must focus on optimizing targeted mass spectrometry protocols or engineering high-affinity multiplexed ELISA arrays capable of accurately quantifying low-abundance epigenetic marks amidst complex serum matrices [81]. Patients exhibiting high H3K18la scores, concomitant with elevated LDH expression and SLC7A11 levels, would be the ideal candidates for lactylation-targeted combinatorial therapies. Establishing such a stratification framework is crucial to move beyond the “one-size-fits-all” approach and realize the promise of precision metabolic oncology (Fig. 9, Right Panel).

## 5 Conclusion and future perspectives: charting the lactyl-landscape

The discovery of histone lactylation has fundamentally bridged the century-old divide between the Warburg effect and the modern understanding of cell fate decisions. For decades, the accumulation of lactate was viewed as a metabolic cul-de-sac—a wasteful byproduct of inefficient energy production. The evidence synthesized in this review argues for a radical inversion of this dogma: lactate is not waste, but a sophisticated “metabolic chronometer.” In our proposed model, lactate-derived epigenetic marks serve to fortify the genome against oxidative stress and ferroptotic death. By converting glycolytic flux into stable epigenetic marks, cancer cells create a physical memory of their metabolic state, one that is specifically engineered to fortify the genome against oxidative stress and ferroptotic death. The “Lactate–Kla–Ferroptosis” axis thus represents a prime example of evolutionary exaptation, where a metabolic liability is repurposed as a master signal for survival.

As we stand on the precipice of translating these insights into the clinic, several profound questions remain that will define the next decade of metabolic oncology. The most pressing challenge is the identification of the specific “readers” of the lactyl code. While we have identified the writers (p300/CBP) and erasers (HDAC1-3, SIRT1-3), the effector proteins that recognize and dock onto lactylated lysines remain largely elusive. Structural biology suggests that proteins containing the YEATS domain, such as AF9 and ENL, which are known to recognize acyl-modifications like crotonylation and acetylation, are prime candidates. Identifying the specific readers that recruit the transcriptional machinery to antioxidant loci like SLC7A11 would provide a novel class of therapeutic targets—"Lactylation Reader Inhibitors"—that could block the downstream consequences of the Warburg effect without interfering with general cellular metabolism.

Furthermore, the exploration of the “non-histone lactylome” represents a vast, uncharted frontier. If the acetylome encompasses thousands of cytosolic proteins, it is statistically improbable that lactylation is confined solely to the nucleus. We hypothesize that key executioners of ferroptosis, such as GPX4, ACSL4, or p53, are direct targets of lactylation. For instance, does the lactylation of p53 prevent its nuclear translocation, thereby abrogating its ability to repress SLC7A11? Does the lactylation of cGAS or STING in the cytosol dampen the innate immune response to mitochondrial DNA leakage? Mapping this non-histone landscape using high-sensitivity mass spectrometry will likely reveal that lactylation is a pervasive post-translational regulator of protein stability and enzymatic function, extending its reach far beyond transcriptional control.

Ultimately, the integration of lactylation biology into the ferroptosis framework offers a rational path to overcome the stagnation in treating refractory solid tumors. We are moving away from the simplistic idea of “starving” the tumor of glucose, which often proves toxic to the host, toward a strategy of “epigenetic reprogramming.” By decoupling the metabolic signal (lactate) from its epigenetic consequence (antioxidant defense), we can strip the tumor of its adaptive shield. In this vulnerable state, the very metabolic features that once defined its aggression—high ROS and iron addiction—become the architects of its destruction. The future of cancer therapy may well lie in turning the Warburg effect against itself, transforming the tumor's metabolic strength into its fatal weakness.

## Funding

This work was supported by the 10.13039/501100007129Shandong Province Natural Science Foundation grants (grant no. ZR2022QH372).

## CRediT authorship contribution statement

**Yali Liu:** Conceptualization, Writing – original draft. **Qiang Yang:** Methodology, Resources, Software, Writing – original draft. **Xiaoqiang Wang:** Software, Visualization, Writing – original draft. **Hai Zhao:** Funding acquisition, Project administration, Supervision, Writing – review & editing.

## Declaration of competing interests

The authors declare that they have no known competing financial interests or personal relationships that could have appeared to influence the work reported in this paper.

## Data Availability

No data was used for the research described in the article.
